# *catena*-Poly[[triaqua­zinc(II)]-μ-1*H*-1,2,4-triazole-3,5-dicarboxyl­ato]

**DOI:** 10.1107/S1600536808023994

**Published:** 2008-08-06

**Authors:** Yan-Yan Sun, Ya-Wen Zhang, Gong Zhang, Lin Cheng

**Affiliations:** a Department of Chemistry and Chemical Engineering, Southeast University, Nanjing, People’s Republic of China; b Department of Chemistry and Chemical Engineering, State Key Laboratory of Coordination Chemistry, Nanjing University, Nanjing, People’s Republic of China

## Abstract

In the title compound, [Zn(C_4_HN_3_O_4_)(H_2_O)_3_]_*n*_, each Zn^II^ atom adopts a distorted octa­hedral coordination geometry, being surrounded by one chelating and one monodentate 1*H*-1,2,4-triazole-3,5-dicarboxyl­ate ligand and three water mol­ecules. Adjacent Zn^II^ cations are linked by a 1*H*-1,2,4-triazole-3,5-dicarboxyl­ate ligand in a μ_2_,κ^3^ fashion to form a chain running along the *c* axis. The crystal packing is stabilized by N—H⋯O, O—H⋯N and O—H⋯O hydrogen bonds.

## Related literature

For related literature, see: Yang *et al.* (2004 ▶); Yin *et al.* (2001 ▶); Tian *et al.* (2003 ▶).
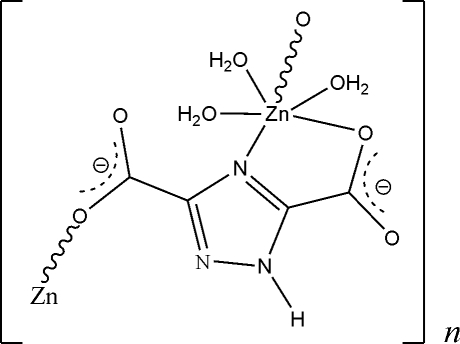


## Experimental

### 

#### Crystal data

[Zn(C_4_HN_3_O_4_)(H_2_O)_3_]
*M* *_r_* = 274.50
Monoclinic, 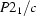

*a* = 10.7388 (11) Å
*b* = 6.6608 (7) Å
*c* = 13.7789 (10) Å
β = 120.384 (6)°
*V* = 850.22 (14) Å^3^
*Z* = 4
Mo *K*α radiation
μ = 2.92 mm^−1^
*T* = 293 (2) K
0.13 × 0.12 × 0.12 mm

#### Data collection

Bruker APEX CCD diffractometer
Absorption correction: multi-scan (*SADABS*; Sheldrick, 2000 ▶) *T*_min_ = 0.703, *T*_max_ = 0.721
4297 measured reflections
1652 independent reflections
1501 reflections with *I* > 2σ(*I*)
*R*_int_ = 0.026

#### Refinement

*R*[*F* ^2^ > 2σ(*F* ^2^)] = 0.034
*wR*(*F* ^2^) = 0.090
*S* = 1.06
1652 reflections
136 parameters
H-atom parameters constrained
Δρ_max_ = 0.53 e Å^−3^
Δρ_min_ = −0.31 e Å^−3^

### 

Data collection: *SMART* (Bruker, 2000 ▶); cell refinement: *SAINT* (Bruker, 2000 ▶); data reduction: *SAINT*; program(s) used to solve structure: *SHELXS97* (Sheldrick, 2008 ▶); program(s) used to refine structure: *SHELXL97* (Sheldrick, 2008 ▶); molecular graphics: *SHELXTL* (Sheldrick, 2008 ▶); software used to prepare material for publication: *SHELXTL*.

## Supplementary Material

Crystal structure: contains datablocks I, global. DOI: 10.1107/S1600536808023994/bt2754sup1.cif

Structure factors: contains datablocks I. DOI: 10.1107/S1600536808023994/bt2754Isup2.hkl

Additional supplementary materials:  crystallographic information; 3D view; checkCIF report

## Figures and Tables

**Table 1:** Hydrogen-bond geometry (Å, °)

*D*—H⋯*A*	*D*—H	H⋯*A*	*D*⋯*A*	*D*—H⋯*A*
N2—H2*A*⋯O1^i^	0.81	1.95	2.723 (3)	161
O1*W*—H1*WA*⋯O2^ii^	0.85	1.85	2.697 (3)	176
O1*W*—H1*WB*⋯O3*W*^iii^	0.85	2.16	2.946 (3)	154
O2*W*—H2*WA*⋯N1^iv^	0.85	2.08	2.925 (4)	172
O2*W*—H2*WB*⋯O3^v^	0.85	2.01	2.848 (3)	170
O3*W*—H3*WA*⋯O3	0.85	1.86	2.708 (3)	174
O3*W*—H3*WB*⋯O4^v^	0.85	1.91	2.753 (3)	174

Symmetry codes: (i) 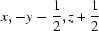; (ii) 
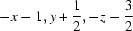
; (iii) 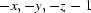; (iv) 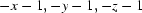; (v) 
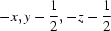
.

**Fig. 1.**
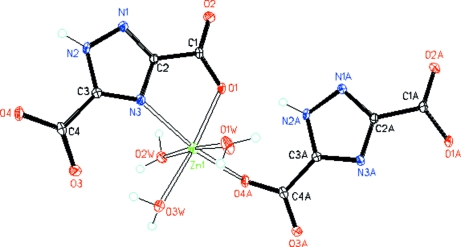
Local coordination environment of the title compound with 30% thermal ellipsoids. Symmetry code: a: x, -1/2 - y, -1/2 + z.

**Fig. 2.**
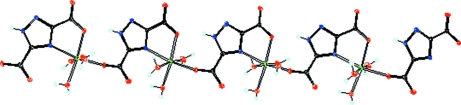
The one-dimensional chain of the title compound.

## supplementary crystallographic information

### Comment

Synthesis and characterization of coordination polymers is of great interest due to the formation of fascinating structures with interesting applications (Yin *et al.* 2001; Yang *et al.* 2004). Among these coordination polymers, one-dimensional chain complexes as important precursors of molecular magnets have attracted wide interest of experimental and theoretical chemists (Tian *et al.* 2003). Herein, we report a new one-dimensional compound [Zn(Htda)(H_2_O)_3_)]*n* (H_3_tda = 1*H*-1,2,4-triazole-3,5-dicarboxylic acid).

The asymmetric unit of the title compound, [Zn(Htda)(H_2_O)_3_)]*n* (H_3_tda = 1*H*-1,2,4-triazole-3,5-dicarboxylic acid), contains a Zn^II^ cation, a Htda anion and three coordinated water molecules. In the compound, the Zn^II^ ion displays a slightly distorted octahedral geometry, being surrounded by one chelating and one monodentate Htda ligands, and three H_2_O molecules. Meanwhile, the adjacent Zn^II^ cations are linked by a µ^3^-Htda ligand to form a one-dimensional chain. The shortest intrachain Zn···Zn distance is 6.936 (4) Å. The chains are further stabilized by N—H···O and O—H···O hydrogen bonds.

### Experimental

A mixture of H_3_tda (0.0157 g, 0.1 mmol), Zn(NO_3_)_2_.6H_2_O (0.0297 g, 0.1 mmol), and water (10 ml) was stirred for 1 h at room temperature, and then filtered. The filtrate was allowed to evaporate slowly at room temperature. After 3 weeks, colorless block crystals were obtained in 30% yield (0.0082 g) based on Zn^II^.

### Refinement

H atoms were located in a difference map but refined as riding with N—H = 0.80 Å and O—H = 0.85 Å and with *U*_iso_(H) = 1.2*U*_iso_(N,O).

### Crystal data

**Table:**

[Zn(C_4_HN_3_O_4_)(H_2_O)_3_]	*F*_000_ = 552
*M**_r_* = 274.50	*D*_x_ = 2.144 Mg m^−^^3^
Monoclinic, *P*2_1_/*c*	Mo *K*α radiation λ = 0.71073 Å
Hall symbol: -P 2ybc	Cell parameters from 805 reflections
*a* = 10.7388 (11) Å	θ = 2.5–28.0º
*b* = 6.6608 (7) Å	µ = 2.92 mm^−^^1^
*c* = 13.7789 (10) Å	*T* = 293 (2) K
β = 120.384 (6)º	Block, colourless
*V* = 850.22 (14) Å^3^	0.13 × 0.12 × 0.12 mm
*Z* = 4	

### Data collection

**Table:**

Bruker APEX CCD diffractometer	1652 independent reflections
Radiation source: fine-focus sealed tube	1501 reflections with *I* > 2σ(*I*)
Monochromator: graphite	*R*_int_ = 0.026
*T* = 293(2) K	θ_max_ = 26.0º
φ and ω scans	θ_min_ = 2.2º
Absorption correction: multi-scan(SADABS; Sheldrick, 2000)	*h* = −12→13
*T*_min_ = 0.703, *T*_max_ = 0.721	*k* = −8→7
4297 measured reflections	*l* = −16→16

### Refinement

**Table:**

Refinement on *F*^2^	Secondary atom site location: difference Fourier map
Least-squares matrix: full	Hydrogen site location: inferred from neighbouring sites
*R*[*F*^2^ > 2σ(*F*^2^)] = 0.034	H-atom parameters constrained
*wR*(*F*^2^) = 0.090	*w* = 1/[σ^2^(*F*_o_^2^) + (0.0485*P*)^2^ + 1.155*P*] where *P* = (*F*_o_^2^ + 2*F*_c_^2^)/3
*S* = 1.06	(Δ/σ)_max_ < 0.001
1652 reflections	Δρ_max_ = 0.53 e Å^−^^3^
136 parameters	Δρ_min_ = −0.31 e Å^−^^3^
Primary atom site location: structure-invariant direct methods	Extinction correction: none

### Special details

**Table:**

Geometry. All e.s.d.'s (except the e.s.d. in the dihedral angle between two l.s. planes) are estimated using the full covariance matrix. The cell e.s.d.'s are taken into account individually in the estimation of e.s.d.'s in distances, angles and torsion angles; correlations between e.s.d.'s in cell parameters are only used when they are defined by crystal symmetry. An approximate (isotropic) treatment of cell e.s.d.'s is used for estimating e.s.d.'s involving l.s. planes.
Refinement. Refinement of *F*^2^ against ALL reflections. The weighted *R*-factor *wR* and goodness of fit *S* are based on *F*^2^, conventional *R*-factors *R* are based on *F*, with *F* set to zero for negative *F*^2^. The threshold expression of *F*^2^ > σ(*F*^2^) is used only for calculating *R*-factors(gt) *etc*. and is not relevant to the choice of reflections for refinement. *R*-factors based on *F*^2^ are statistically about twice as large as those based on *F*, and *R*- factors based on ALL data will be even larger.

### Fractional atomic coordinates and isotropic or equivalent isotropic displacement parameters (Å^2^)

**Table:**

	*x*	*y*	*z*	*U*_iso_*/*U*_eq_	
Zn1	−0.21910 (4)	−0.31001 (6)	−0.54678 (3)	0.01968 (16)	
C1	−0.5300 (4)	−0.3318 (5)	−0.6300 (2)	0.0150 (6)	
C2	−0.4615 (3)	−0.2580 (5)	−0.5104 (2)	0.0138 (6)	
C3	−0.2985 (3)	−0.1691 (5)	−0.3493 (3)	0.0158 (6)	
C4	−0.1588 (4)	−0.1138 (5)	−0.2453 (3)	0.0188 (7)	
N1	−0.5320 (3)	−0.2264 (4)	−0.4566 (2)	0.0164 (6)	
N2	−0.4261 (3)	−0.1687 (4)	−0.3544 (2)	0.0166 (6)	
H2A	−0.4471	−0.1452	−0.3071	0.020*	
N3	−0.3183 (3)	−0.2244 (4)	−0.4487 (2)	0.0155 (5)	
O1	−0.4393 (2)	−0.3607 (4)	−0.66272 (18)	0.0200 (5)	
O2	−0.6604 (3)	−0.3595 (4)	−0.68401 (19)	0.0258 (6)	
O3	−0.0468 (3)	−0.1174 (5)	−0.2503 (2)	0.0327 (6)	
O4	−0.1676 (2)	−0.0634 (4)	−0.16134 (18)	0.0227 (5)	
O1W	−0.2327 (3)	−0.0209 (4)	−0.60933 (19)	0.0305 (6)	
H1WA	−0.2626	0.0338	−0.6731	0.037*	
H1WB	−0.1556	0.0484	−0.5776	0.037*	
O2W	−0.1842 (2)	−0.6059 (4)	−0.47695 (19)	0.0231 (5)	
H2WA	−0.2630	−0.6585	−0.4892	0.028*	
H2WB	−0.1227	−0.6151	−0.4072	0.028*	
O3W	−0.0099 (2)	−0.2331 (4)	−0.42261 (18)	0.0199 (5)	
H3WA	−0.0169	−0.1900	−0.3675	0.024*	
H3WB	0.0497	−0.3301	−0.3975	0.024*	

### Atomic displacement parameters (Å^2^)

**Table:**

	*U*^11^	*U*^22^	*U*^33^	*U*^12^	*U*^13^	*U*^23^
Zn1	0.0174 (2)	0.0265 (2)	0.0153 (2)	−0.00014 (16)	0.00829 (18)	0.00025 (15)
C1	0.0181 (16)	0.0154 (15)	0.0108 (15)	−0.0001 (12)	0.0069 (13)	0.0002 (12)
C2	0.0149 (16)	0.0154 (14)	0.0108 (14)	0.0004 (12)	0.0062 (13)	0.0004 (12)
C3	0.0148 (16)	0.0180 (15)	0.0142 (15)	−0.0001 (12)	0.0070 (13)	0.0000 (12)
C4	0.0200 (17)	0.0205 (16)	0.0114 (15)	−0.0017 (14)	0.0047 (14)	−0.0023 (12)
N1	0.0147 (14)	0.0214 (14)	0.0127 (13)	−0.0021 (11)	0.0066 (11)	−0.0020 (10)
N2	0.0192 (14)	0.0234 (14)	0.0097 (13)	0.0000 (11)	0.0090 (12)	−0.0015 (10)
N3	0.0149 (14)	0.0185 (13)	0.0117 (13)	−0.0020 (11)	0.0058 (11)	−0.0016 (10)
O1	0.0192 (12)	0.0314 (13)	0.0105 (11)	−0.0023 (10)	0.0083 (10)	−0.0029 (9)
O2	0.0139 (12)	0.0414 (15)	0.0172 (12)	−0.0040 (11)	0.0042 (10)	−0.0087 (11)
O3	0.0151 (13)	0.0615 (18)	0.0201 (13)	−0.0055 (12)	0.0079 (11)	−0.0142 (12)
O4	0.0230 (13)	0.0337 (13)	0.0122 (11)	−0.0086 (11)	0.0095 (10)	−0.0049 (10)
O1W	0.0323 (15)	0.0272 (13)	0.0200 (12)	−0.0081 (11)	0.0045 (11)	0.0055 (10)
O2W	0.0187 (12)	0.0288 (13)	0.0187 (12)	−0.0025 (10)	0.0072 (10)	0.0037 (10)
O3W	0.0187 (12)	0.0252 (12)	0.0170 (11)	0.0015 (10)	0.0098 (10)	−0.0006 (10)

### Geometric parameters (Å, °)

**Table:**

Zn1—O1W	2.085 (2)	C3—C4	1.505 (5)
Zn1—O3W	2.085 (2)	C4—O3	1.241 (4)
Zn1—O4^i^	2.096 (2)	C4—O4	1.252 (4)
Zn1—O1	2.106 (2)	N1—N2	1.342 (4)
Zn1—O2W	2.141 (2)	N2—H2A	0.8053
Zn1—N3	2.177 (3)	O4—Zn1^ii^	2.096 (2)
C1—O2	1.223 (4)	O1W—H1WA	0.8499
C1—O1	1.278 (4)	O1W—H1WB	0.8500
C1—C2	1.508 (4)	O2W—H2WA	0.8500
C2—N1	1.316 (4)	O2W—H2WB	0.8500
C2—N3	1.348 (4)	O3W—H3WA	0.8500
C3—N3	1.328 (4)	O3W—H3WB	0.8500
C3—N2	1.336 (4)		
			
O1W—Zn1—O3W	86.17 (10)	N2—C3—C4	123.4 (3)
O1W—Zn1—O4^i^	92.88 (10)	O3—C4—O4	125.8 (3)
O3W—Zn1—O4^i^	97.55 (9)	O3—C4—C3	118.0 (3)
O1W—Zn1—O1	91.02 (10)	O4—C4—C3	116.2 (3)
O3W—Zn1—O1	172.75 (9)	C2—N1—N2	102.3 (3)
O4^i^—Zn1—O1	89.25 (9)	C3—N2—N1	110.9 (3)
O1W—Zn1—O2W	174.71 (10)	C3—N2—H2A	131.1
O3W—Zn1—O2W	89.22 (9)	N1—N2—H2A	118.0
O4^i^—Zn1—O2W	85.14 (9)	C3—N3—C2	103.4 (3)
O1—Zn1—O2W	93.86 (9)	C3—N3—Zn1	147.1 (2)
O1W—Zn1—N3	93.49 (10)	C2—N3—Zn1	109.04 (19)
O3W—Zn1—N3	95.09 (9)	C1—O1—Zn1	118.15 (19)
O4^i^—Zn1—N3	166.20 (10)	C4—O4—Zn1^ii^	139.4 (2)
O1—Zn1—N3	78.40 (9)	Zn1—O1W—H1WA	137.2
O2W—Zn1—N3	89.51 (9)	Zn1—O1W—H1WB	116.3
O2—C1—O1	127.2 (3)	H1WA—O1W—H1WB	93.4
O2—C1—C2	119.3 (3)	Zn1—O2W—H2WA	111.1
O1—C1—C2	113.4 (3)	Zn1—O2W—H2WB	115.7
N1—C2—N3	114.6 (3)	H2WA—O2W—H2WB	108.8
N1—C2—C1	124.5 (3)	Zn1—O3W—H3WA	105.7
N3—C2—C1	120.9 (3)	Zn1—O3W—H3WB	115.1
N3—C3—N2	108.8 (3)	H3WA—O3W—H3WB	106.3
N3—C3—C4	127.8 (3)		

Symmetry codes: (i) *x*, −*y*−1/2, *z*−1/2; (ii) *x*, −*y*−1/2, *z*+1/2.

### Hydrogen-bond geometry (Å, °)

**Table:**

*D*—H···*A*	*D*—H	H···*A*	*D*···*A*	*D*—H···*A*
N2—H2A···O1^ii^	0.81	1.95	2.723 (3)	161
O1W—H1WA···O2^iii^	0.85	1.85	2.697 (3)	176
O1W—H1WB···O3W^iv^	0.85	2.16	2.946 (3)	154
O2W—H2WA···N1^v^	0.85	2.08	2.925 (4)	172
O2W—H2WB···O3^vi^	0.85	2.01	2.848 (3)	170
O3W—H3WA···O3	0.85	1.86	2.708 (3)	174
O3W—H3WB···O4^vi^	0.85	1.91	2.753 (3)	174

Symmetry codes: (ii) *x*, −*y*−1/2, *z*+1/2; (iii) −*x*−1, *y*+1/2, −*z*−3/2; (iv) −*x*, −*y*, −*z*−1; (v) −*x*−1, −*y*−1, −*z*−1; (vi) −*x*, *y*−1/2, −*z*−1/2.
